# Pekinenin E Inhibits the Growth of Hepatocellular Carcinoma by Promoting Endoplasmic Reticulum Stress Mediated Cell Death

**DOI:** 10.3389/fphar.2017.00424

**Published:** 2017-06-29

**Authors:** Lu Fan, Qingling Xiao, Yanyan Chen, Gang Chen, Jinao Duan, Weiwei Tao

**Affiliations:** ^1^School of Medicine and Life Sciences, Nanjing University of Chinese MedicineNanjing, China; ^2^School of Basic Biomedical Science, Nanjing University of Chinese MedicineNanjing, China; ^3^Jiangsu Key Laboratory for High Technology Research of TCM Formulae and Jiangsu Collaborative Innovation Center of Chinese Medicinal Resources Industrialization, Nanjing University of Chinese MedicineNanjing, China

**Keywords:** pekinenin E, hepatocarcinoma, ER stress, apoptosis, cell cycle arrest

## Abstract

Hepatocellular carcinoma (HCC) is a malignant primary liver cancer with poor prognosis. In the present study, we report that pekinenin E (PE), a casbane diterpenoid derived from the roots of *Euphorbia pekinensis*, has a strong antitumor activity against human HCC cells both *in vitro* and *in vivo*. PE suppressed the growth of human HCC cells Hep G2 and SMMC-7721. In addition, PE-mediated endoplasmic reticulum (ER) stress caused increasing expressions of C/EBP homologous protein (CHOP), leading to apoptosis in HCC cells both *in vitro* and *in vivo*. Inhibition of ER stress with CHOP small interfering RNA or 4-phenyl-butyric acid partially reversed PE-induced cell death. Furthermore, PE induced S cell cycle arrest, which could also be partially reversed by CHOP knockdown. In all, these findings suggest that PE causes ER stress-associated cell death and cell cycle arrest, and it may serve as a potent agent for curing human HCC.

## Introduction

Hepatocellular carcinoma (HCC) is the third leading cause of cancer death (Forner et al., 2012). Several kinds of treatments might be beneficial for HCC, such as surgical resection, liver transplantation, percutaneous ethanol injection, transarterial chemoembolization, and transarterial radioembolization. Moreover, patients with HCC usually exhibit poor tolerance to systemic chemotherapy because of their abnormal liver function (Yang and Roberts, 2010). Many important molecules and pathways contribute to liver carcinogenesis, for example, receptor tyrosine kinases, Wnt β-catenin signaling (Lu et al., 2015), the ubiquitin–proteasome system (Chen et al., 2016), epigenetic DNA modification (promoter methylation and histone acetylation) (Yuan et al., 2011; Ko et al., 2016) and the PI3K–AKT–mTOR pathway (Zhang et al., 2013). Starley et al. (2010) reported that endoplasmic reticulum (ER) stress was also involved in HCC development. These considerations highlight the importance of developing new treatments based on the understanding of pathogenic molecular mechanisms.

Pekinenin E (PE) is a casbane diterpenoid derived from the roots of *Euphorbia pekinensis*, which has been used to treat edema, tuberculosis, and liver cirrhosis ascites in Chinese traditional medicine for many years (Shao et al., 2011). Our previous study has shown that casbane diterpenoids isolated from the roots of *E. pekinensis* had a strong antiproliferative activity against multiple cancer types (Tao et al., 2013). However, to our knowledge, the underlying mechanism of the anticancer effect of PE remains to be investigated.

This study revealed that PE inhibited the growth of HCC by promoting ER stress mediated cell death.

## Materials and Methods

### Materials

Antibodies against ATF4 (#11815), phospho-eIF2α (#3398), C/EBP homologous protein (CHOP) (#2895), GRP78 (#3177), Cyclin A2 (#4656), CDK2 (#2546), Cleaved Caspase-9 (#9505), Caspase-9 (#9502), eIF2α (#5324), and PARP (#9532) were purchased from Cell Signaling Technology (Beverly, MA, United States). Annexin V-fluorescein isothiocyanate (FITC)/propidium iodide (PI) kit was purchased from BD Biosciences (San Jose, CA, United States). Antibodies against ATF6α (sc-22799) was purchased from Santa Cruz Biotechnology (Santa Cruz, CA, United States). Cleaved PARP (ab32064) and β-actin (ab-8226) antibodies were purchased from Abcam (Cambridge, MA, United States). Dimethyl sulfoxide (DMSO) was obtained from Sigma Chemical Co. (St. Louis, MO, United States). Protease Inhibitor Cocktail was obtained from Roche Technology (Basel, Switzerland). PE was isolated and purified in our laboratory (Tao et al., 2013).

### Cell Culture and PE Treatment

Human HCC cells Hep G2, SMMC-7721, human lung epithelial cells BEAS-2B and rat heart myoblast cells H9c2 were purchased from the Shanghai Institute of Cell Biology (Shanghai, China), maintained in Dulbecco’s modified Eagle’s medium (DMEM; Gibco, Grand Island, NY, United States) supplemented with 10% fetal bovine serum (Gibco), 100 U/ml penicillin, and 100 mg/ml streptomycin, and incubated under a humidified 5% (v/v) CO_2_ atmosphere at 37°C. PE was dissolved in DMSO to a concentration of 30 mM (stock solution) and stored at -20°C.

### Mice

Forty male BALB/c athymic nude mice (5–6 weeks old, 15–18 g) were purchased from the Experimental Animal Center of Jiangsu Province (Nanjing, China). Animal welfare and experimental procedures were conducted in accordance with the Provision and General Recommendation of Chinese Experimental Animals Administration Legislation and were approved by Animal Ethics Committee of Nanjing University of Chinese Medicine. 5 × 10^6^ SMMC-7721 cells were suspended in 100 μl phosphate-buffered saline (PBS) and injected subcutaneously into the right flank regions of each mouse. Two weeks later, the mice were divided randomly into four groups: control group, PE 12.5 mg/kg group, PE 25 mg/kg group, PE 50 mg/kg group (*n* = 10 per group). PE dissolved in 0.5 ml PBS was intragastrically administered for continuous 7 days. Body weight and tumor volumes were measured and recorded every 2 days. After the last treatment of PE, the mice were sacrificed. The tumor tissues were excised for pending tests.

### MTT Assay

Cells viability was measured using MTT solution (4 mg/ml in PBS) and incubated for 4 h at 37°C. After removing incubation medium, the purple formazan crystals were dissolved in 200 μl of DMSO for 5 min. The absorbance at 570 nm was determined in each well on an automated microplate spectrophotometer (Sunrise, Tecan, Austria).

### Reverse Transcription and Quantitative PCR

Total RNA was isolated from cells as described (Fan et al., 2014a). Subsequently, cDNA was synthesized from 1 μg total RNA by reverse transcription using oligo (dT) (Hidalgo et al., 2001). Quantitative PCR (Q-PCR) was carried out with the ABI Prism 7000 sequence detection system (Applied Biosystems, Foster City, CA, United States) using SYBR Green I dye (Biotium, Inc., Hayward, CA, United States). The primer sequences used were as follows (5′–3′): *GRP78* (NM_005347.4) sense, CATCACGCCGTCCTATGTCG and antisense CGTCAAAGACCGTGTTCTCG; *GRP94* (NM_003299.2) sense, GCTGACGATGAAGTTGATGTGG and antisense CATCCGTCCTTGATCCTTCTCTA; *CHOP* (NM_001195053.1) sense, GGAAACAGAGTGGTCATTCCC and antisense, CTGCTTGAGCCGTTCATTCTC; *ATF4* (NM_001675.4) sense TGAAGGAGTTCGACTTGGATGCC and antisense CAGAAGGTCATCTGGCATGGTTTC; *GAPDH* (NM_001289745.1) sense GAGTCAACGGATTTGGTCGT and antisense TTGATTTTGGAGGGATCTCG.

### Flow Cytometry

For apoptosis detection, cells were resuspended in Annexin-V binding buffer, and stained with 2.5 μl of Annexin V-FITC and 2 μl of PI for 10 min at room temperature in the dark, followed by cytometric analysis (EPICS XL, Beckman Coulter, Fullerton, CA, United States) within 30 min of staining. Samples were analyzed using a FACSCalibur flow cytometer (Fan et al., 2014a).

### Western Blot

Cell extracts and immunoblots were performed as described (Fan et al., 2014b).

### Electron Microscopy

The protocol for transmission electron microscopy has been reported previously (Fan et al., 2014b).

### Immunofluorescence Microscopy

Cells on glass cover slips were washed with PBS and fixed with 4% paraformaldehyde for 30 min and permeabilized with 0.1% Triton X-100 for 20 min. Then cells were blocked with bovine serum albumin (5% in PBS) for 1 h at room temperature, followed by incubating with antibody against CHOP overnight at 4°C. Slides were washed three times with PBS, and incubated with Alexa-488 conjugated goat anti mouse (Invitrogen, Carlsbad, CA, United States) for 1 h at room temperature. Nuclei were counterstained with 2 μg/ml 4′,6-diamidino-2-phenylindole for 1 min. The fluorescent signals were observed under a mercury lamp (Olympus, Tokyo, Japan).

### Small Interfering RNA Transfection

CHOP small interfering RNA (siRNA) sequence used in SMMC-7721 cells was: 5′-UUCAUCUGAAGACAGGACCUCUUGC-3′. Luciferase siRNA was used as described before (Wang et al., 2011). Cells were transfected with luciferase siRNA or CHOP siRNA using Lipofectamine 2000 (Life Technologies, Carlsbad, CA, United States) for 24 h according to the manufacturer’s instructions (Fan et al., 2014a). Then cells were treated with either 30 μM PE or 0.1% DMSO as control for another 24 h.

### TUNEL Staining

TUNEL staining was performed using the *in situ* apoptosis detection TUNEL kit (Roche, Switzerland) following manufacturer’s instructions as described (Fan et al., 2014a).

### Statistical Analysis

Data were expressed as the mean ± standard deviation (SD) of three independent experiments. One-way ANOVA and *post hoc* tests were applied when there are more than two groups in the independent variable. Two-way ANOVA followed by *post hoc* tests were used to examine the effects of two variables. *P*-value <0.05 is regarded as statistically significant.

## Results

### PE Inhibits Human Hepatocellular Carcinoma Cell Growth

**FIGURE 1:**
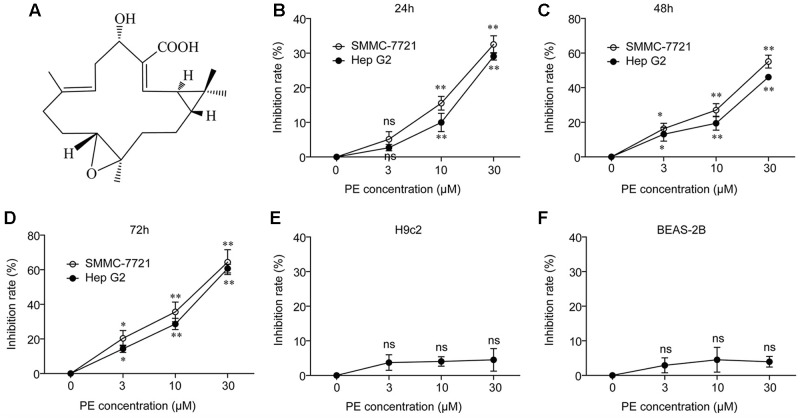
PE inhibits human hepatocellular carcinoma cell growth without affecting normal control cells. **(A)** Chemical structure of PE. **(B–D)** Hep G2 and SMMC-7721 cells were incubated with PE at various concentrations for 24, 48, and 72 h, respectively. The cell viability was determined by MTT assay. Values were expressed as mean ± SD of three independent experiments. ^∗^*P* < 0.05, ^∗∗^*P* < 0.01 versus control cells cultured with 0.1% DMSO by one-way ANOVA and *post hoc* tests. **(E,F)** The effect of PE on cell viability in non-malignant cells was determined by MTT assay. H9c2 and BEAS-2B cells were incubated with PE at various concentrations for 48 h. Values were expressed as mean ± SD of three independent experiments.

The structure of PE is presented in **Figure 1A**. In order to assess the effect of PE on HCC cells, MTT assay was performed. Human HCC cell line Hep G2 and SMMC-7721 cells were treated with various concentrations of PE for 24, 48, and 72 h, respectively. The results revealed that PE showed a dose-dependent inhibition of human HCC cells (**Figures 1B–D**). However, PE did not affect human lung epithelial cells BEAS-2B and rat heart myoblast cells H9c2 (**Figures 1E,F**).

### PE Induces Apoptosis in Hepatocellular Carcinoma Cell Lines

**FIGURE 2:**
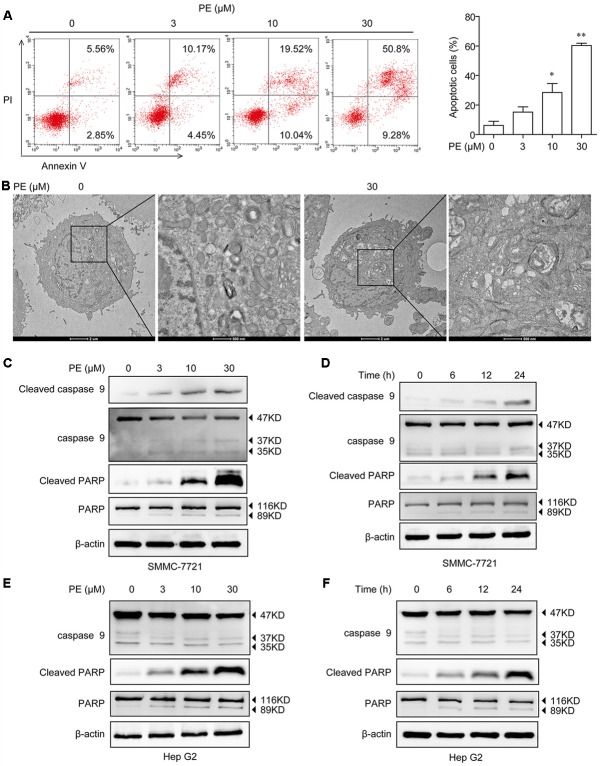
Effects of PE on cell apoptosis in hepatocellular carcinoma cell lines. **(A)** SMMC-7721 cells were seeded in six-well plate and incubated with 0, 3, 10, and 30 μM PE for 24 h. The apoptosis of SMMC-7721 cells was determined by Annexin V/PI staining. Values were expressed as mean ± SD of three independent experiments. ^∗^*P* < 0.05, ^∗∗^*P* < 0.01 versus control cells cultured with 0.1% DMSO by one-way ANOVA and *post hoc* tests. **(B)** Representative transmission electron micrographs of SMMC-7721 cells treated with or without PE. After treated with 30 μM PE for 24 h, SMMC-7721 cells exhibited the characteristic ultrastructural morphology of apoptosis, such as vacuolation in cytoplasm, chromatin condensation, and apoptotic body. **(C)** SMMC-7721 cells were incubated with various concentrations of PE for 24 h, immunoblots against Cleaved Caspase-9, Caspase-9, Cleaved PARP, PARP, and β-actin were detected. β-Actin was taken as control. Blots are representative of three independent experiments. **(D)** SMMC-7721 cells were incubated with 30 μM PE for 0, 6, 12, and 24 h, Cleaved Caspase-9, Caspase-9, Cleaved PARP, PARP, and β-actin were determined by western blotting. β-Actin was taken as control. Blots are representative of three independent experiments. **(E)** Hep G2 cells were incubated with various concentrations of PE for 24 h; immunoblots against Caspase-9, Cleaved PARP, PARP, and β-actin were detected. β-Actin was taken as control. Blots are representative of three independent experiments. **(F)** Hep G2 cells were incubated with 30 μM PE for 0, 6, 12, and 24 h, Caspase-9, Cleaved PARP, PARP, and β-actin were determined by western blotting. β-Actin was taken as control. Blots are representative of three independent experiments.

In order to detect the mechanism of the inhibition of PE toward human HCC cells, the apoptosis of SMMC-7721 cells was determined by Annexin V/PI staining. As shown in **Figure 2A**, PE dose-dependently induced apoptosis of SMMC-7721 cells, and approximately 60.08% of SMMC-7721 cells underwent apoptosis after treatment with 30 μM PE for 24 h. Next, SMMC-7721 cells treated with 30 μM PE or not were fixed for transmission electron microscopy to demonstrate the ultrastructural morphology. We found that SMMC-7721 cells treated with 30 μM PE exhibited the characteristic morphology of apoptosis, such as vacuolation in cytoplasm, chromatin condensation, and apoptotic body (**Figure 2B**). Subsequently, the cell extracts of SMMC-7721 and Hep G2 cells were prepared for immunoblotting after treated with PE. PE increased the protein levels of Cleaved Caspase-9 and Cleaved PARP in both dose- and time-dependent manners (**Figures 2C–F** and Supplementary Figure 1).

### PE Induces ER Stress in Hepatocellular Carcinoma Cell Lines

**FIGURE 3:**
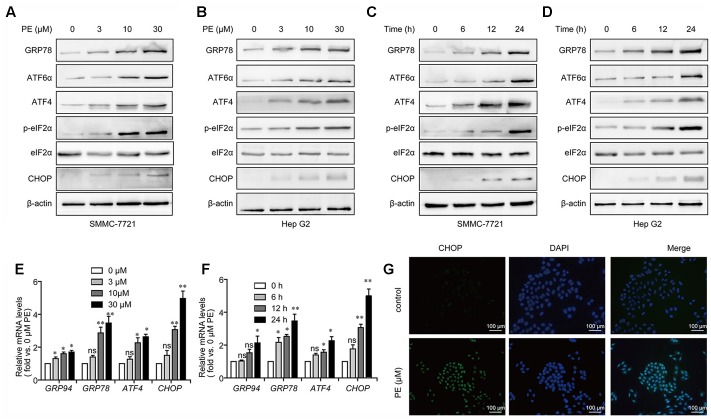
PE induces ER stress in hepatocellular carcinoma cell lines. **(A,B)** Representative immunoblots against GRP78, ATF6α, ATF4, p-eIF2α, eIF2α, CHOP, and β-actin from cell lysates of SMMC-7721 and Hep G2 treated with PE (0, 3, 10, and 30 μM) for 24 h. β-Actin was taken as control. Blots are representative of three independent experiments. **(C,D)** SMMC-7721 and Hep G2 cells were treated by 30 μM PE for 0, 6, 12, and 24 h. Immunoblots against GRP78, ATF6α, ATF4, p-eIF2α, eIF2α, CHOP, and β-actin from cell lysates of SMMC-7721 and Hep G2 were detected. β-Actin was taken as control. Blots are representative of three independent experiments. **(E)** Representative mRNA levels of *GRP94*, *GRP78*, *ATF4*, and *CHOP* from SMMC-7721 cell treated with PE (0, 3, 10, and 30 μM) for 24 h. *GAPDH* was used as control. Values were expressed as mean ± SD of three independent experiments. ^∗^*P* < 0.05, ^∗∗^*P* < 0.01 versus control cells cultured with 0.1% DMSO by one-way ANOVA and *post hoc* tests. **(F)** Representative mRNA levels of *GRP94*, *GRP78*, *ATF4*, and *CHOP* from cell lysates of SMMC-7721 treated with 30 μM PE for 0, 6, 12, and 24 h. *GAPDH* was taken as control. Values were expressed as mean ± SD of three independent experiments. ^∗^*P* < 0.05, ^∗∗^*P* < 0.01 versus control cells cultured with 0 h by one-way ANOVA and *post hoc* tests. **(G)** Fluorescence microscopic images of the SMMC-7721 cells stained with CHOP in both control and PE treated cells. Scale bars, 100 μm.

Next, we examined the effects of PE in PE-treated SMMC-7721 and Hep G2 cells in relation to unfolded protein response (UPR). After 24-h exposure to PE, a significant increase in the phosphorylation of eIF2α was found. Moreover, PE treatment increased GRP78, ATF6α, ATF4, and CHOP expression in both dose- and time-dependent manners (**Figures 3A–D** and Supplementary Figure 2). Q-PCR data also validated our findings that PE up-regulated the mRNA levels of *ATF4*, *GRP94*, *GRP78*, and *CHOP* (**Figures 3E,F**) in SMMC-7721 cells. Subsequently, fluorescence images clearly showed the increase of CHOP in 30 μM PE treated SMMC-7721 cell nuclei compared to the untreated ones (**Figure 3G**). These findings indicate that PE induces ER stress in HCC cells.

### PE Induces ER Stress-Mediated Cell Death in Hepatocellular Carcinoma Cell Lines

**FIGURE 4:**
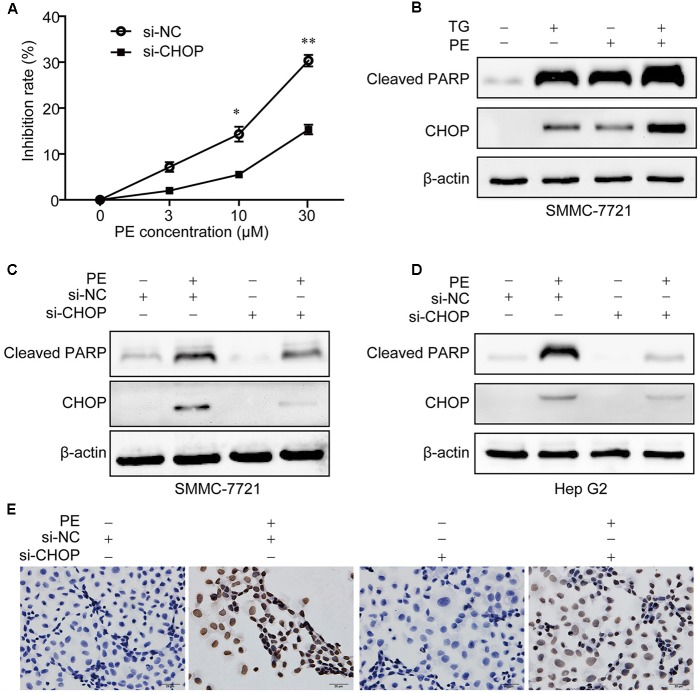
PE induces ER stress mediated apoptosis in hepatocellular carcinoma cell lines. **(A)** SMMC-7721 cells were seeded in six-well plates for 12 h, transfected with Luc siRNA or CHOP siRNA for 24 h followed by incubation with 0, 3, 10, and 30 μM PE for another 24 h. Cell growth of three independent experiments was shown. Values were expressed as mean ± SD of three independent experiments. ^∗^*P* < 0.05, ^∗∗^*P* < 0.01 versus PE group by two-way ANOVA and *post hoc* tests. **(B)** SMMC-7721 cells were incubated with PE for 24 h in the absence or presence of TG (1 μM). The protein levels of Cleaved PARP and CHOP were determined by western blotting. β-Actin was taken as control. Blots are representative of three independent experiments. **(C–E)** SMMC-7721 or Hep G2 cells were transfected with Luc siRNA or CHOP siRNA for 24 h followed by incubation with 30 μM PE for another 24 h. **(C,D)** The protein levels of Cleaved PARP and CHOP were determined by western blotting. β-Actin was taken as control. Blots are representative of three independent experiments. **(E)** SMMC-7721 cell apoptosis was detected using TUNEL staining.

To assess the role of ER stress in PE-induced apoptosis, CHOP was knocked down in SMMC-7721 and Hep G2 cells. As shown in **Figure 4A**, CHOP siRNA significantly reduced PE-induced repression of cell growth. Addition of the commercially available ER stress inducer, thapsigargin (TG), resulted in an enhancement of CHOP and Cleaved PARP expression in PE treated SMMC-7721 cells, which were also increased on PE treatment (**Figure 4B**). Further investigation showed that knockdown of CHOP greatly decreased CHOP and Cleaved PARP expression in SMMC-7721 and Hep G2 cells (**Figures 4C,D**). Next, 4-phenyl-butyric acid (4-PBA), an inhibitor of ER stress (Ozcan et al., 2006), was used to test whether it could ameliorate the effects of PE *in vitro*. Our results showed that 4-PBA reverses the increase of Cleaved PARP and CHOP induced by PE (Supplementary Figure 3). Furthermore, knocking down of CHOP expression with siRNA reversed PE-induced apoptosis (**Figure 4E**). Taken together, these results suggest that PE-induced SMMC-7721 cell apoptosis is partly mediated through the ER stress, at least.

### PE Induces S Cell-Cycle Arrest via ER Stress

**FIGURE 5:**
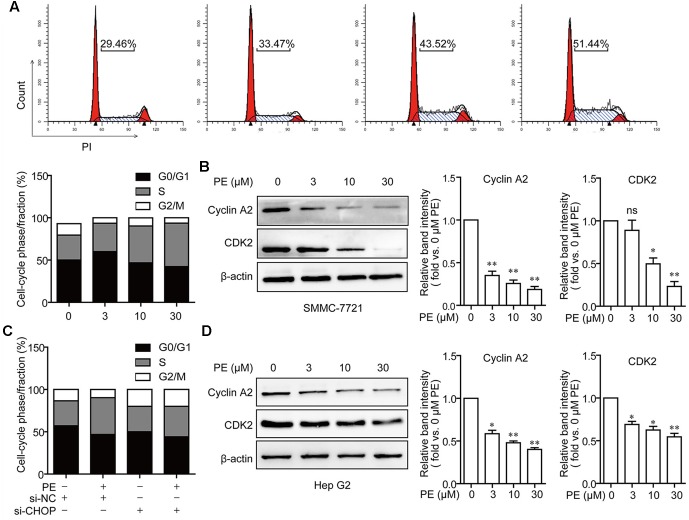
PE induces S cell cycle arrest via ER stress in SMMC-7721 cells. **(A)** S cell cycle arrest in PE-treated SMMC-7721 cells. Cells were incubated with indicated concentrations of PE for 24 h. Propidium iodide staining and flow cytometry were used to determine the proportion of cells in various phases of the cell cycle. **(B)** Representative immunoblots against Cyclin A2, CDK2, and β-actin from SMMC-7721 cell lysates after treated with PE (0, 3, 10, and 30 μM) for 24 h. β-Actin was taken as control. Blots are representative of three independent experiments. Values were expressed as mean ± SD of three independent experiments. ^∗^*P* < 0.05, ^∗∗^*P* < 0.01 versus control cells cultured with 0.1% DMSO by one-way ANOVA and *post hoc* tests. **(C)** The effects of CHOP knockdown on cell cycle arrest. SMMC-7721 cells were transfected with Luc siRNA or CHOP siRNA for 24 h, followed by incubation with 30 μM of PE for another 24 h. **(D)** Representative immunoblots against Cyclin A2, CDK2, and β-actin from cell lysates of Hep G2 treated with PE (0, 3, 10, and 30 μM) for 24 h. β-Actin was taken as control. Blots are representative of three independent experiments. Values were expressed as mean ± SD of three independent experiments. ^∗^*P* < 0.05, ^∗∗^*P* < 0.01 versus control cells cultured with 0.1% DMSO by one-way ANOVA and *post hoc* tests.

Measurements of DNA content showed that PE dose-dependently increased the percentage of cells in S phase (**Figure 5A**). After treatment with 30 μM PE, the percentage of cells at S phase (51.44%) was significantly increased compared to that of the control group (29.46%). Moreover, the protein levels of CDK2 and Cyclin A2 were decreased in a dose-dependent manner in PE treated SMMC-7721 and Hep G2 cells (**Figures 5B,D**). Furthermore, knocking down of CHOP suppressed S cell-cycle arrest in PE-treated SMMC-7721 cells (**Figure 5C**). These results demonstrate that PE induces S cell-cycle arrest via ER stress.

### PE Suppresses the Growth of Hepatocellular Carcinoma in BALB/c Athymic Nude Mice with Apoptosis Induction

**FIGURE 6:**
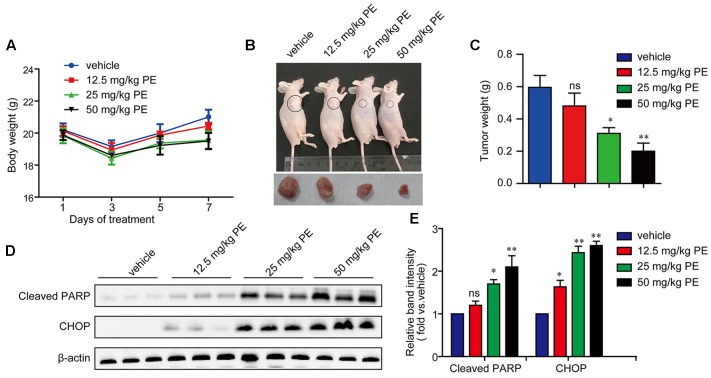
PE inhibits the growth of hepatocellular carcinoma in BALB/c athymic nude mice. 5 × 10^6^ cells were injected subcutaneously into right flank regions of each mouse. PBS (vehicle control) or various concentrations of PE were administrated every day. **(A)** Body weight during administration of PE. **(B)** Representative pictures of tumor from mice per group were shown. **(C)** Tumor weight after 7 days administration of PE. Values were expressed as mean ± SD. ^∗^*P* < 0.05, ^∗∗^*P* < 0.01 versus vehicle by one-way ANOVA and *post hoc* tests. **(D,E)** Immunoblots against Cleaved PARP and CHOP in tumor samples were determined. β-actin was performed as control. Blots are representative of the 10 mice per group. Values were expressed as mean ± SD. ^∗^*P* < 0.05, ^∗∗^*P* < 0.01 versus vehicle by one-way ANOVA and *post hoc* tests.

5 × 10^6^ SMMC-7721 cells suspended in 100 μl PBS were injected subcutaneously into the right flank regions of each mouse. Two weeks later when the tumors began to enlarge (about 500 mm^3^), the mice were divided randomly into four groups: control group, PE 12.5 mg/kg group, PE 25 mg/kg group, and PE 50 mg/kg group (*n* = 10 per group). PE did not have significant effect on the body weight, as well as the weight of the liver or spleen (**Figure 6A** and Supplementary Figure 4). The administration of PE inhibited tumor growth compared with vehicles in a dose-dependent manner (**Figure 6B**). When the tumors were removed, the average weight of tumors from PE-treated mice at the 50 mg/kg dose was threefold less than the vehicle-tumors (**Figure 6C**). The protein levels of CHOP and Cleaved PARP in the tumor tissues removed were greatly elevated in the PE treatment group (**Figures 6D,E**).

## Discussion

So far, there is only surgery that could offer a high rate of complete responses in HCC patients (Bruix and Sherman, 2011). Systemic chemotherapy has marginal activity with frequent toxic effects, without survival benefit, and agents such as tamoxifen, octreotide, or antiandrogens are completely ineffective (Bruix and Sherman, 2011; Forner et al., 2012). PE, a casbane diterpenoid isolated from *E. pekinensis*, was found to have a very strong antitumor activity in various cancer types (Tao et al., 2013). Interestingly, we highlighted a strong inhibition of SMMC-7721 and Hep G2 cells by PE without any influence on non-malignant cells (**Figures 1B–F**). Moreover, PE induces apoptosis in SMMC-7721 cells (**Figure 2**). But a clear understanding of the exact molecular mechanisms of PE is required to specifically designate.

Elevated ER stress and the UPR are detected in many cancers, including HCC (Wang et al., 2010). ER stress has been shown to generate pre-oncogenic cells that lead to HCC under conditions of a high-fat-diet-induced inflammatory environment (Nakagawa et al., 2014), indicating an oncogenic role for ER stress. The role of ER stress in HCC was also reported in the context of sorafenib treatment (Yi et al., 2012). Shuda et al. (2003) reported that ATF6 was involved in HCC development. Thus, the induction of ER stress represents a new therapeutic strategy for killing tumor cells (Wang and Kaufman, 2014). In the current study, PE was found to induce an ER stress response typified by CHOP induction. Meanwhile, an elevation of GRP78/BIP, ATF6α, ATF4, and p-eIF2α were detected (**Figure 3**). These results implied that PE induced ER stress through PERK-p-eIF2α-ATF4 pathway and ATF6 pathway (**Figure 3**). In contrast, PE did not activate XBP1, indicating that PE had little or no effect on IRE1–XBP1 pathway (data not shown). When ER stress is excessive and homeostasis is not restored, the UPR triggers apoptosis to eliminate the damaged cells (Hetz, 2012). Cao et al. (2014) reported that prolonged UPR activation induces apoptosis, mainly through activation of the PERK–eIF2α–ATF4–CHOP pathway. CHOP, which was encoded by the gene DDIT3, could induce the expression of proapoptotic genes, which triggers apoptosis during ER stress. When DDIT3 was knockdown, ER stress causes less protein aggregation in the ER and reduces oxidative stress and apoptosis (Marciniak et al., 2004; Malhotra et al., 2008; Wang and Kaufman, 2014). In this respect, we found that inhibition of ER stress with CHOP siRNA or ER stress inhibitor, 4-PBA, partially reversed PE-induced cell death and induction of the cleavage of PARP (**Figure 4** and Supplementary Figure 3). These results demonstrate that PE-induced HCC cell apoptosis is partly mediated through ER stress, at least.

Diehl (2002) and Ho et al. (2014) reported that dysregulation of the cell cycle was involved in tumorigenesis. In this study, we found that PE promoted S cell cycle arrest in SMMC-7721 cells (**Figure 5**). These results could be due to the induction of ER stress by PE. Cyclin A and CDK2 could form a complex for the promotion of the cell cycle, regulating cell cycle progression from S-phase to G2/M (Liu et al., 2013). Consistent with this, we found that PE decreased Cyclin A2 and CDK2 expression in SMMC-7721 cells (**Figures 5B,D**). Moreover, CHOP knockdown significantly blocked PE-induced S cell cycle arrest (**Figure 5C**). Thus, we deduce that ER stress contribute to PE-induced S cell cycle arrest.

These data were further confirmed *in vivo* using SMMC-7721 xenograft mouse mode. We found that PE treatment dose-dependently inhibited tumor growth and reduced tumor weight in a SMMC-7721 xenograft model, but had little effect on the body weight (**Figure 6**). These results indicate that PE has relatively low toxicity in animals. In this study, PE showed potent anti-HCC activity both *in vitro* and *in vivo*. These results implicate its clinical therapy potential.

**FIGURE 7:**
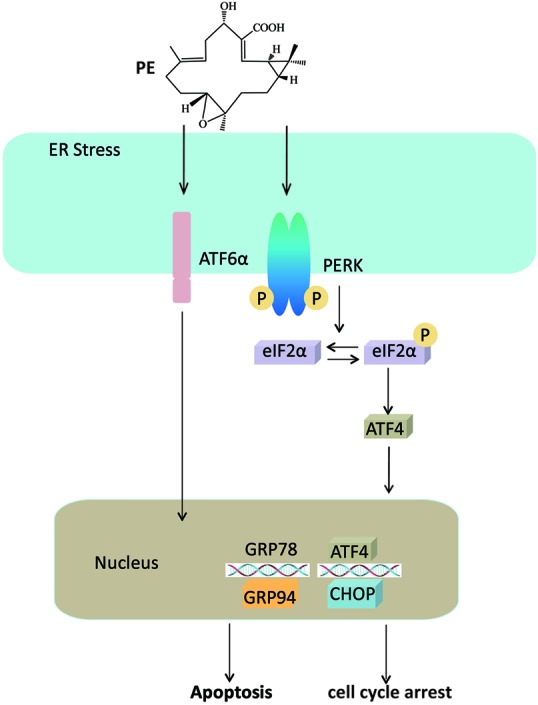
Mechanism for PE’s effects on hepatocellular carcinoma cells. PE induces ER stress through PERK-p-eIF2α-ATF4 pathway and ATF6 pathway, leading to the overexpression of CHOP, which triggers apoptosis and cell cycle arrest.

In summary, as illustrated in **Figure 7**, our current study highlights that PE treatment potentiates the ER stress mediated pathway in human HCC cells. We also offer the first *in vivo* evidence for PE’s strong antitumor effect. Thus, PE treatment could represent a new therapeutic strategy for curing human HCC.

## Author Contributions

LF and WT contributed to study conception and design. LF, QX, and YC contributed to acquisition, analysis, and/or interpretation of data. LF, QX, GC, JD, and WT performed drafting/revision of the work for intellectual content and context. WT contributed to final approval and overall responsibility for the published work.

## Conflict of Interest Statement

The authors declare that the research was conducted in the absence of any commercial or financial relationships that could be construed as a potential conflict of interest.
