# Supplementary material for: Pekinenin E Inhibits the Growth of Hepatocellular Carcinoma by Promoting Endoplasmic Reticulum Stress Mediated Cell Death
Source: Front Pharmacol. 2017 Jun 29;8:424. doi: 10.3389/fphar.2017.00424 (PMC5489557; doi:10.3389/fphar.2017.00424)
Supplement: Supplementary file 1 [file Data_Sheet_1.doc]

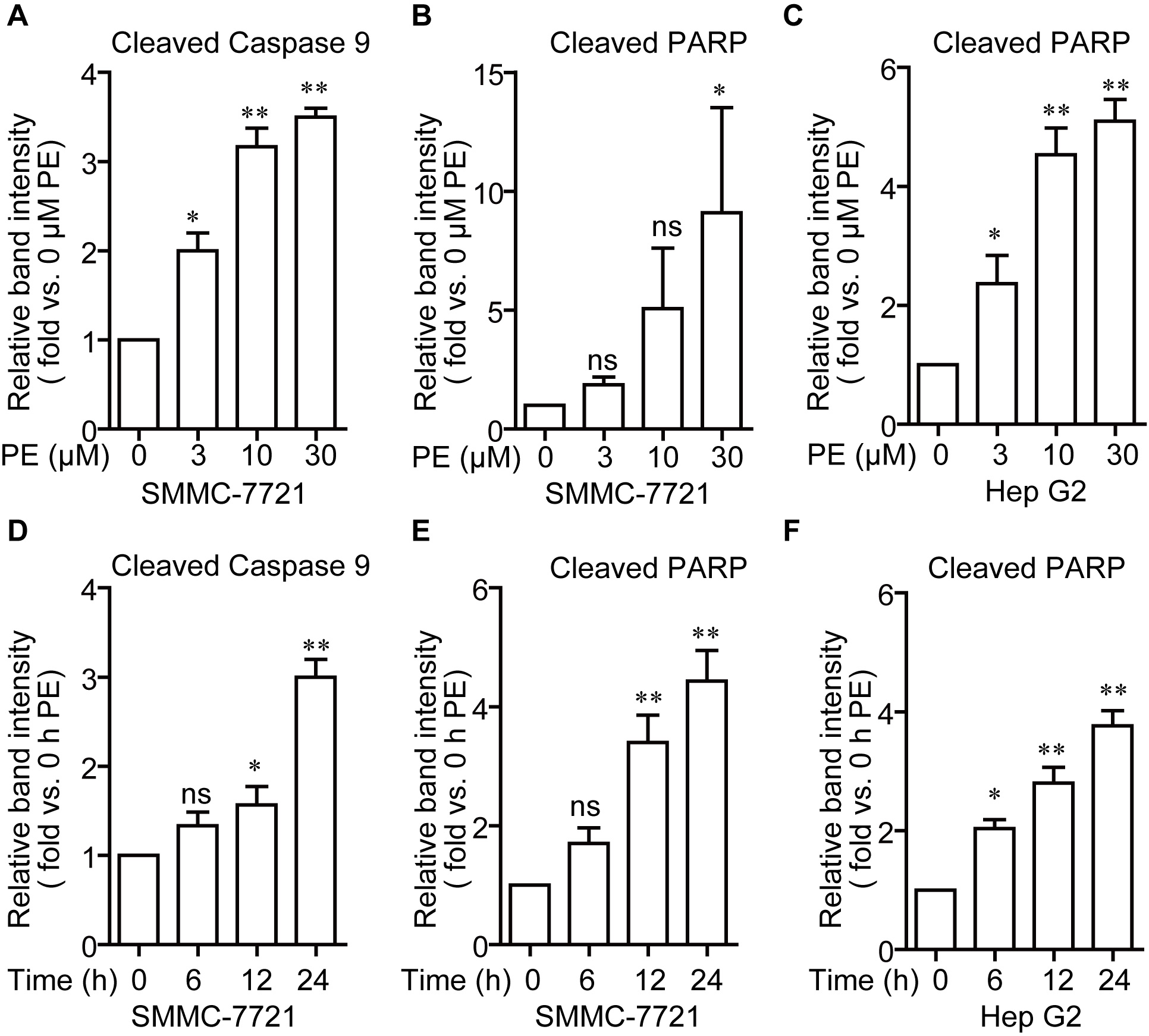


Supplementary Fig. 1. (A-B) SMMC-7721 cells were treated by different concentrations of PE for 24 h, Cleaved caspase 9, caspase 9, Cleaved PARP, PARP and β-actin were determined by western blotting. β-actin was used as control. Relative band intensity of Cleaved caspase 9 and Cleaved PARP were analyzed with Image J software. Values were expressed as mean ± SD of three independent experiments. *P < 0.05, **P <0.01 versus control cells cultured with 0.1% DMSO by One-way ANOVA analysis and post-hoc tests. (C) Hep G2 cells were treated by different concentrations of PE for 24 h, caspase 9, Cleaved PARP, PARP and β-actin were determined by western blotting. β-actin was used as control. Relative band intensity of Cleaved PARP was analyzed with Image J software. Values were expressed as mean ± SD of three independent experiments. *P < 0.05, **P <0.01 versus control cells cultured with 0.1% DMSO by One-way ANOVA analysis and post-hoc tests. (D-E) SMMC-7721 cells were treated by 30 μM PE for 0, 6, 12, 24 h, Cleaved caspase 9, caspase 9, Cleaved PARP, PARP and β-actin were determined by western blotting. β-actin was used as control. Relative band intensity of Cleaved caspase 9 and Cleaved PARP were analyzed with Image J software. Values were expressed as mean ± SD of three independent experiments. *P < 0.05, **P <0.01 versus control cells cultured with 0 h by One-way ANOVA analysis and post-hoc tests. (F) Hep G2 cells were treated by 30 μM PE for 0, 6, 12 and 24 h, caspase 9, Cleaved PARP, PARP and β-actin were determined by western blotting. β-actin was used as control. Relative band intensity of Cleaved PARP was analyzed with Image J software. Values were expressed as mean ± SD of three independent experiments. *P < 0.05, **P <0.01 versus control cells cultured with 0 h by One-way ANOVA analysis and post-hoc tests.


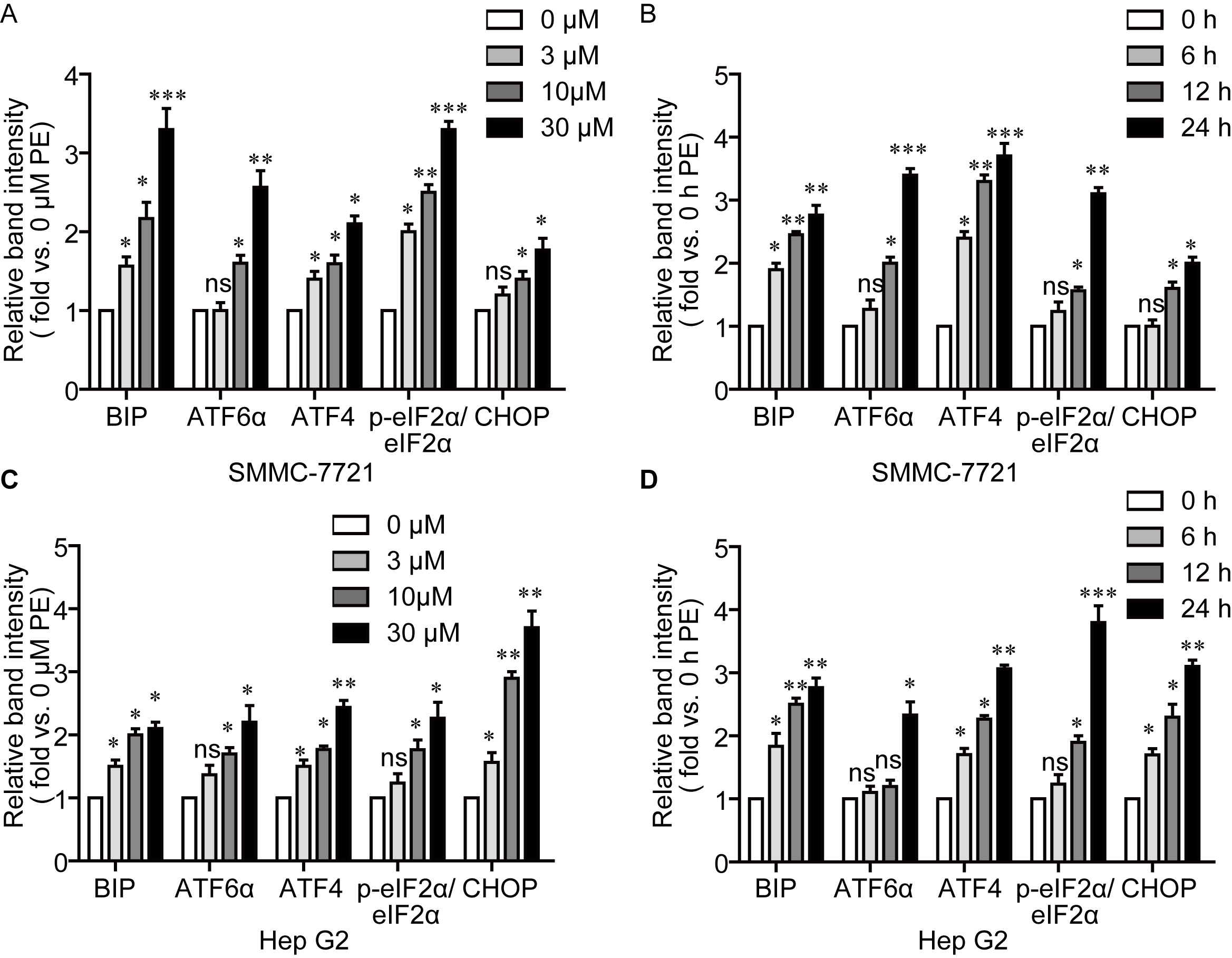


Supplementary Fig. 2. (A, C) Representative immunoblots against BIP, ATF6α, ATF4, p-eIF2α, eIF2α, CHOP and β-actin from cell lysates of SMMC-7721 and Hep G2 treated with PE (0, 3, 10 and 30 μM) for 24 hours. β-actin was used as control. Relative band intensity was analyzed with Image J software. Relative p-eIF2α level was analyzed with western blotting and data were presented as ratio of p-eIF2α to total eIF2α in the form of grayscale value. Values were expressed as mean ± SD of three independent experiments. *P < 0.05, **P <0.01, ***P <0.001versus control cells cultured with 0.1% DMSO by One-way ANOVA analysis and post-hoc tests. (B, D) SMMC-7721 and Hep G2 cells were treated by 30 μM PE for 0, 6, 12, 24 hours, immunoblots against BIP, ATF6α, ATF4, p-eIF2α, eIF2α, CHOP and β-actin from cell lysates of SMMC-7721 and Hep G2 were detected. β-actin was used as control. Relative band intensity was analyzed with Image J software. Relative p-eIF2α level was analyzed with western blotting and data were presented as ratio of p-eIF2α to total eIF2α in the form of grayscale value. Values were expressed as mean ± SD of three independent experiments. *P < 0.05, **P <0.01, ***P <0.001 versus control cells cultured with 0 h by One-way ANOVA analysis and post-hoc tests.


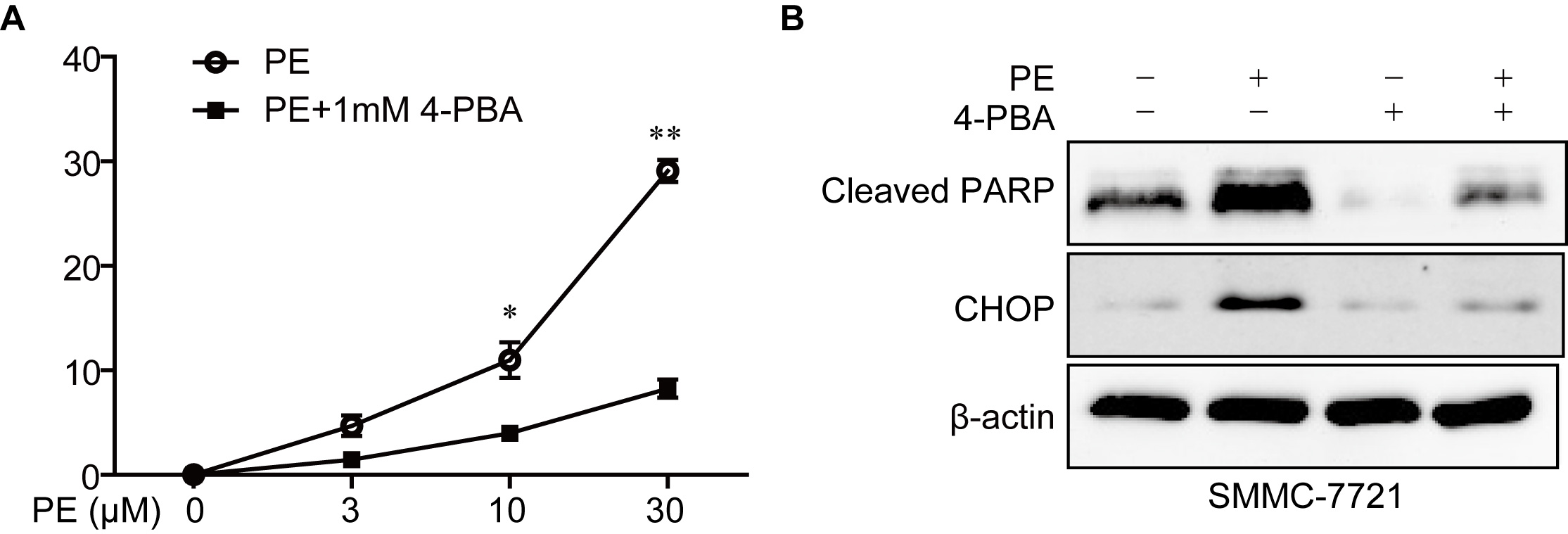


Supplementary Fig. 3. SMMC-7721 cells were treated with PE for 24 h in the absence or presence of 4-PBA (1 mM). (A) Cell growth of three independent experiments was shown. Cell number were counted by Typan blue staining and normalized with 0.1% DMSO. Values were expressed as mean ± SD of three independent experiments. *P < 0.05, **P <0.01 versus PE group by Two-way ANOVA analysis and post-hoc tests. (B) The protein levels of Cleaved PARP and CHOP were determined with immunoblotting. β-actin was performed as a loading control. Blots are representative of three independent experiments.


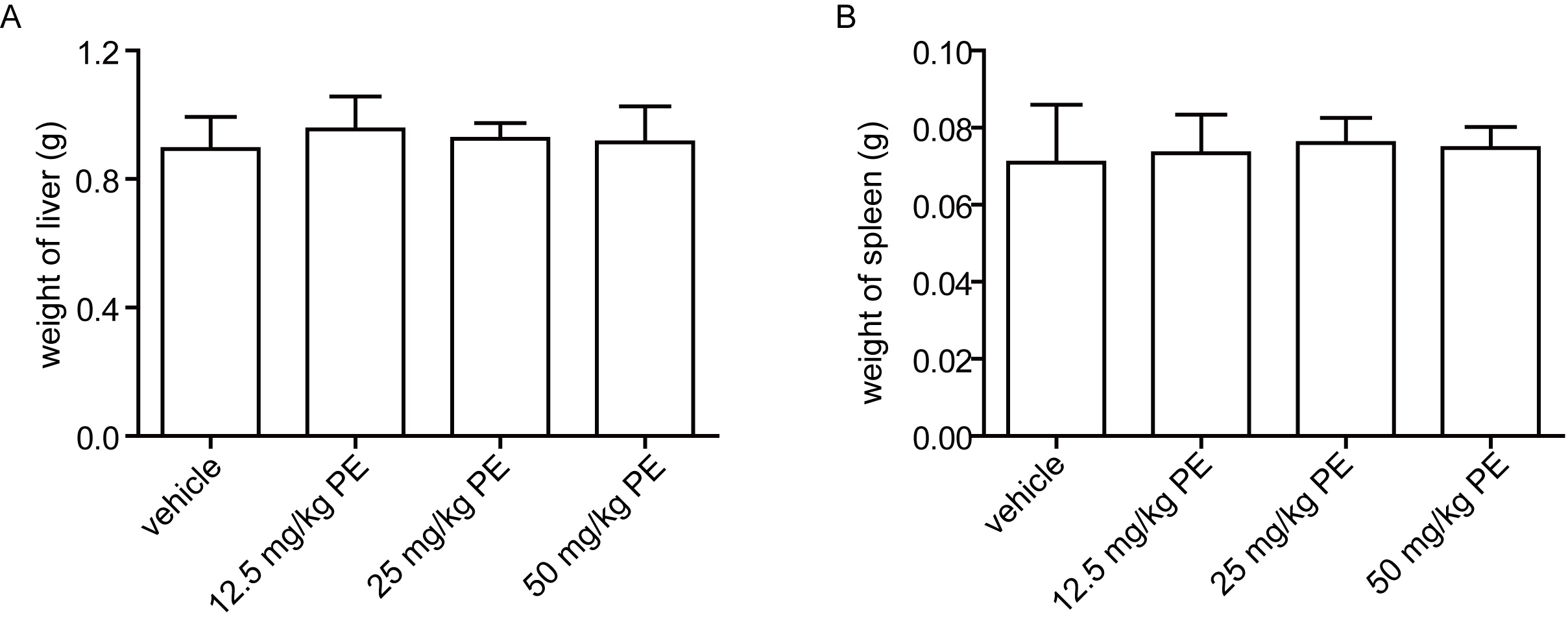


Supplementary Fig. 4. (A-B). Weight of liver and spleen.
